# Normoxic and Hyperoxic Cardiopulmonary Bypass in Congenital Heart Disease

**DOI:** 10.1155/2014/678268

**Published:** 2014-09-18

**Authors:** Amir Mokhtari, Martin Lewis

**Affiliations:** ^1^Bristol Heart Institute, University of Bristol, Bristol BS2 8HW, UK; ^2^Department of Cardiology, School of Clinical Sciences, Faculty of Medicine and Dentistry, University of Bristol, Bristol BS10 5NB, UK; ^3^Department of Cardiac Surgery, Leeds General Infirmary, Leeds Teaching Hospitals NHS Trust, Leeds LS1 3EX, UK; ^4^Bristol Royal Infirmary, University Hospitals Bristol NHS Foundation Trust, Bristol BS2 8HW, UK; ^5^School of Clinical Sciences, University of Bristol, 69 St Michael's Hill, Bristol BS2 8DZ, UK

## Abstract

Cyanotic congenital heart disease comprises a diverse spectrum of anatomical pathologies. Common to all, however, is chronic hypoxia before these lesions are operated upon when cardiopulmonary bypass is initiated. A range of functional and structural adaptations take place in the chronically hypoxic heart, which, whilst protective in the hypoxic state, are deleterious when the availability of oxygen to the myocardium is suddenly improved. Conventional cardiopulmonary bypass delivers hyperoxic perfusion to the myocardium and is associated with cardiac injury and systemic stress, whilst a normoxic perfusate protects against these insults.

## 1. Introduction

**Figure 1:**
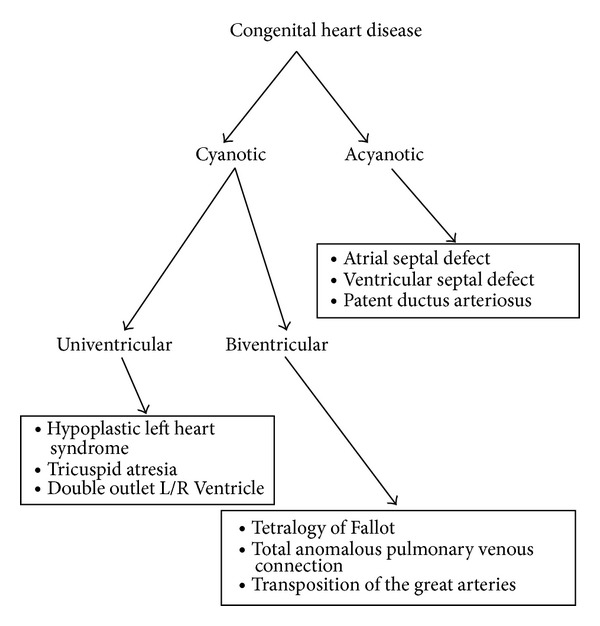
Schematic representation of a classification system for Congenital Heart Disease, with examples of typical lesions from each category. Acyanotic disease makes up the majority of cases of congenital abnormalities, and a large proportion goes silent and undiagnosed. The cyanotic lesions will undergo surgical repair.

As a consequence of advances made in the fields of paediatric cardiac surgery, anaesthesia, and perfusion science, an increasing number of surgical repairs of congenital cardiac abnormalities are being carried out each year. In fact the field of paediatric cardiac surgery has progressed so far that virtually no lesion is considered “inoperable” [1]. These congenital cardiac abnormalities fall into two broad groups; those causing cyanosis through intra- or extracardiac right to left sided shunts, or those which are not acyanotic (Figure 1). The cyanotic type is, by definition, associated with chronic hypoxia, as well as with consequent malnutrition and growth failure [2], and the uncorrected has outcomes that are universally worse than the acyanotic group.

The cyanotic group may be further divided along many lines, but a significant classification from an operative perspective is into those patients with a physiologically functional double-ventricle circulation and those with only a single functioning ventricle. Evidence is emerging that those patients with univentricular pathologies are more vulnerable to deleterious systemic and myocardial effects from standard “hyperoxic” cardiopulmonary bypass (CPB) during and after operative treatment of these pathologies than those patients with biventricular pathologies [3]. However, both of these groups show evidence of end-organ injury as well as systemic inflammation and stress when they undergo conventional hyperoxic CPB [4–6].

Therefore, this review seeks to examine the mechanisms by which hearts in patients with cyanotic-type circulations adapt to chronic hypoxia, the mechanisms underlying so-called reoxygenation injury from animal studies, and also the current clinical evidence showing the existence of this phenomenon perioperatively.

## 2. Cardiac Metabolic and Adaptive Responses to Hypoxia

**Figure 2:**
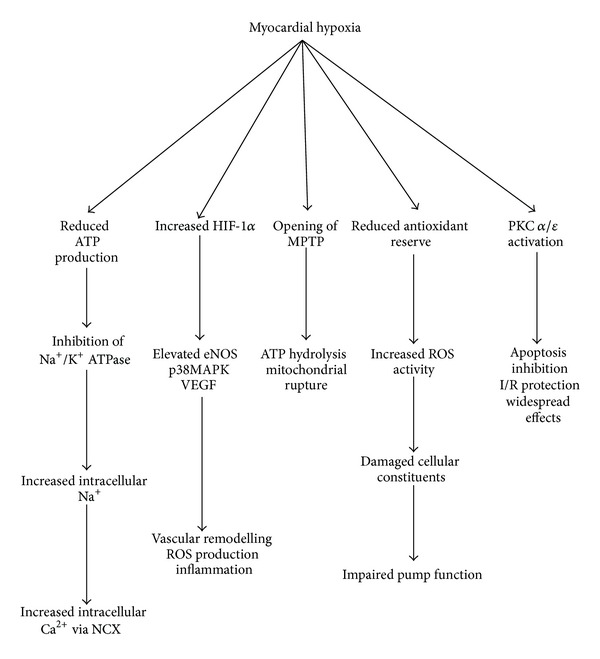
Schematic representation of selected consequences of hypoxia in the cardiomyocyte. Shown here are five different downstream chains of events initiated under hypoxic conditions in the cardiomyocyte. These pathways have significant horizontal interaction which synergises and amplify the outcome, and not all of these events occur synchronously or in all contexts. A reduction in ATP production and MPTP opening alongside PKC activation are early consequences of acute ischaemia, whilst the effectors of HIF-1 occur sometime later.

Hypoxia is an imbalance between tissue oxygen demand and oxygen supply in the context of normal tissue perfusion. A number of processes, both adaptive and maladaptive, take place under hypoxic conditions, and these contribute to the risk of reoxygenation injury under hyperoxic conditions during CPB (Figure 2). These processes prime the myocyte for particular responses when perfusion with oxygen and respiratory fuel is reintroduced and so influence the eventual degree of reoxygenation injury.

### 2.1. Hypoxic Adaptations

Significant changes in the myocyte gene expression profile occur in the chronically hypoxic heart. Hypoxia inducible factor 1 (HIF-1) is a transcription factor whose significance during hypoxic states is increasingly evident [7]. During normoxia, constitutively expressed HIF-1*α* is degraded by HIF prolyl-hydroxylase. However in hypoxic conditions, HIF prolyl-hydroxylase is inhibited which results in HIF-1*α* accumulation in the cytoplasm and translocation to the nucleus [8, 9]. This leads to activation of many genes including, for instance, erythropoietin (EPO) and VEGF [10, 11]—these targets produce changes in the processes of angiogenesis, vascular remodelling, erythropoiesis, ROS production, and inflammation. In the chronically hypoxic heart, as might be expected, HIF-1*α* levels are significantly increased, to a level proportionate to the degree of hypoxia. In addition, under the influence of HIF-1*α*, phosphorylated p38MAPK levels as well as eNOS and VEGF levels increase as would be expected [12]. These are adaptive changes to chronic hypoxia and collectively produce a heart which at a genomic and ultrastructural level is profoundly altered.

Hypoxia also alters the intracellular distribution of protein kinase C (PKC) [13] isoforms, including *α* but also *ε* [14]. Under hypoxic conditions, these isoforms are more abundant in particulate fractions than soluble, signifying a translocation from cytosolic to membrane-bound compartments and activation of PKC activity. Mammalian PKC *α* is known to play important roles in the control of cell proliferation and exerts antiapoptotic properties [15, 16], whilst *ε* has for some time been closely associated with responses to ischaemia and protection from I/R injury.

In normal physiological conditions most of the cardiomyocyte ATP production is from mitochondrial oxidative phosphorylation, which even under physiological conditions leads to a small amount of reactive oxygen species (ROS) production. However this form of ATP production is diminished during hypoxia due to reduced oxidation of fatty acid and carbohydrate [17, 18] and consequently processes that are ATP dependent such as ion exchange by the Na^+^/K^+^ ATPase are inhibited. Therefore cardiac metabolism switches partially to anaerobic metabolic pathways, which results in a metabolic acidosis as the degree of tissue hypoxia progresses. This acidosis leads to increased intracellular Na^+^ via Na^+^/H^+^ exchanger (NHE) and, consequently, elevated levels of intracellular Ca^2+^ (through the Na^+^/Ca^2+^ exchanger (NCX)) [18]. These processes are interesting, in the context of investigating the ideal way of treating paediatric cyanotic heart disease; however they are limited by the fact that these experimental studies examine a state of complete* anoxia*, rather than the pathophysiological state of hypoxia.

### 2.2. Reperfusion and Reoxygenation: Intracellular Events

ROS are intrinsic byproducts of the mitochondrion through oxidative phosphorylation. There are cellular mechanisms that counterbalance the physiological accumulation of small amounts of reactive oxygen species; these include enzymes such as catalase and glutathione peroxidase that ultimately reduce hydrogen peroxide and other organic peroxides into less reactive species. Superoxide dismutase (SOD) isoforms may also form part of this endogenous myocardial antioxidant mechanism, albeit upstream, since they facilitate hydrogen peroxide formation from superoxide [19]. However, in the hypoxic situation, whether acute or chronic, there is a lower antioxidant reserve due to increased ROS production. This imbalance leads to interaction of these ROS with many cellular constituents, including nucleic acids, lipid, and proteins, resulting in cell damage and death [20–22].

Other mechanisms for production of ROS have also been noted; the most significant of these is perhaps the Fenton pathway [23]. This describes a process where hydrogen peroxide undergoes catalytic oxidation by Fe^2+^ ions into a hydroxyl radical and hydroxide ion, and the resultant Fe^3+^ is then reoxidised back to ferrous iron by a further molecule of hydrogen peroxide, to leave byproducts of a superoxide radical and hydrogen ion. As a whole, the disproportionation with hydrogen peroxide results in two different reactive oxygen species being formed, which then go on to participate in secondary oxidation of cellular components. Additionally, Beckman et al. [24] described another possible mechanism of oxidant injury when cytotoxic reactive oxygen species are formed as a result of interaction between superoxide anion (O_2_^∙−^) and nitric oxide (NO). In the experimental setting, with cyanosis and a reduction in endogenous myocardial antioxidants, free radical generation during reoxygenation is augmented [25]. In the cardiomyocyte, this alters ion channel flux leading to reductions in pump function and ultimately contractile impairment [20]. A further burst of ROS activity occurs immediately during reperfusion [26]. This effect, coupled with the initial ROS flux from the hypoxic period, causes both primary impairment and damage to the myocyte, as well as increasing the likelihood of mitochondrial pore opening.

Reperfusion and reoxygenation results in opening of the mitochondrial permeability transition pore (MPTP) [27]. Under conditions of reperfusion with attendant oxidative stress, or chronic congestive heart failure as might be seen in more advanced or severe forms of congenital heart disease, the levels of intracellular and mitochondrial Ca^2+^ rise. In this situation, this pore, a nonselective 1.5 kDa channel in the inner mitochondrial membrane, opens, which leads to swelling of the mitochondrion, metabolic uncoupling, ATP hydrolysis, and ultimately cell death. The probability of pore opening is enhanced by other conditions that are present in the myocyte made ischaemic and subsequently reperfused, adenine nucleotide depletion, high levels of inorganic phosphate, and high concentrations of ROS all synergise to stabilise the open pore state. Slightly confusing this picture, however, is the fact that in some animal studies, chronic hypoxia is protective against both MPTP opening and increased ROS flux on reoxygenation. The details of this are not well established, but it does mean that the effects of MPTP opening on reoxygenation injury are not straightforward.

It is believed that the results of surgical repair of cyanotic heart defects are complicated by both local myocardial and wider systemic multiorgan damage as a consequence in part of acute reoxygenation at the time of institution of cardiopulmonary bypass with elevated oxygen content, followed by myocardial ischaemia required to arrest the heart with the subsequent reperfusion [28]. In order to further investigate these phenomena, a number of experimental and clinical studies have been performed to delineate their nature.

## 3. Reoxygenation and Reperfusion Injury: Animal and Clinical Studies

The existence of reoxygenation injury due to a high P_a_O_2_ in the field of resuscitation is well established [29]. There has been much interest in widening the scope of these findings, and there is now strong clinical and experimental evidence indicating that immature hearts have a marked increase in tolerance to ischaemia and a greater resistance against the damaging effects of ischaemia and reperfusion injury (I/R) than mature hearts [30–35]. However, some conflicting clinical and animal studies at intermediate age groups suggests that the postnatal, developing myocardium, is more susceptible to reperfusion injury compared to the adult heart [36–40].

These studies have tended to compare adult heart with* one selected* developmental age group. Differences in the choice of age have contributed to some of the conflicting results. This issue has been highlighted by Awad et al. [41] who investigated the vulnerability of the intact rat heart during different stages of development (4, 7, 14, and 21 days, and adult). Their work and later work from others [42] demonstrated that the recovery of developing rat heart following ischaemia and reperfusion changes during maturation and appears to follow a bell-shaped relationship. Clinical research in paediatric surgery also supports the view that vulnerability to I/R changes during postnatal development [43].

There are some proposed explanations for these ontogenetic changes such as lower energy demand in the neonatal group, higher glycogen levels and glycolytic flux [44, 45], a greater anaerobic capacity, and alterations in Ca^2+^ handling plus ROS production [46]. All of these processes accommodate the shift from a hypoxic in utero environ to a normoxic ex utero one. However, the precise mechanisms behind this varying resistance to oxygen deprivation in neonatal myocardium have still not been clearly defined [47, 48].

Cyanosis, chronic hypoxia, remains the most common preoperative physiological stress in paediatric cardiac patients [1, 49] as well as the single largest cause of mortality [47, 48]. In the clinical setting, it seems as though patients with cyanotic congenital heart defects have severely diminished myocardial protection [1], as well as persistently impaired ventricular function following corrective surgery and a higher rate of morbidity and mortality compared to nonhypoxaemic patients [50–52].

In comparison to the acyanotic population, cyanotic patients have an inferior protective cardioplegic protection effect even with comparatively shorter ischaemic intervals [53]. One possible explanation could be the fact that, in children with decreased pulmonary blood flow, that is, those with significant shunts producing cyanosis, there is an increased bronchial collateral drainage to the left heart that can noticeably compromise intraoperative myocardial protection [1]. Further, in cyanotic pathologies, even the degree of cyanosis can affect the cardiac metabolic reserve. For instance, Imura and coworkers reported worse reperfusion injury and clinical outcome in cyanotic patients when compared to acyanotic patients [54], whilst Najm and coworkers studied forty-eight patients who underwent a repair of Tetralogy of Fallot (TOF) and noticed a correlation between preoperative hypoxia and the degree of ventricular dysfunction [55]. They divided the patients into 3 groups. The first group consisted of 14 patients with preoperative arterial oxygen saturation of 90% to 100%; oxygen saturation of sixteen patients in a second group was between 80% and 89% and the remaining eighteen patients in the third group had an oxygen saturation of 79% or less. They reported that even before any surgical intervention the group with O_2_ saturation of ≤79% had the most impaired ventricular function. They also reported lowest ATP levels preischaemic and 15 minutes following ischaemic arrest. They concluded that the degree of cyanosis has adverse effects on myocardial ATP levels as well as worse clinical outcome.

Further evidence confirming an alteration in oxidative metabolism in the clinical setting came from Del Nido and colleagues [51], this time looking at perioperative changes. This group obtained myocardial biopsies from 16 children undergoing surgical repair of the tetralogy of Fallot as well as 20 adults with coronary artery disease. They showed that TOF patients have a further reduction in adenosine triphosphate and increased lactate not only during ischaemia, but also during reperfusion. They therefore suggested that the limitation to oxidative metabolism exacerbates reperfusion injury as a mechanism of myocardial damage in the setting of cyanotic heart disease.

Changes to oxidative phosphorylation are not the only pathophysiological difference in patients with cyanotic heart disease. Teoh et al. [56] theorised that those patients with cyanotic disease are more susceptible to ROS induced injury. They compared myocardial biopsies of patients with the tetralogy of Fallot to acyanotic adults and reported that patients with tetralogy of Fallot are at higher risk of oxygen-derived free radical injury during cardiac surgery. Examining genetic influences on vulnerability, we [57] investigated myocardial gene profile from right ventricular biopsy of 20 TOF patients undergoing surgical correction. 11 of these patients were cyanotic, whilst 9 were acyanotic. We discovered that the cyanotic population had a widespread increase in gene expression associated with apoptosis and remodelling alongside reduced expression of genes associated with myocardial contractility. As might have been predicted from a theoretical approach to these conditions, those patients with cyanotic disease do demonstrate increased vulnerability both to hypoxia and to reperfusion injury; this seems to be multifactorial, with influences from alterations in oxidative phosphorylation, ROS flux and genomic/transcriptomic variation.

## 4. Experimental Evidence for Hyperoxic Injury

Numerous studies have been carried out to examine the effect of exposure to higher levels of oxygen in hypoxic animal models. Corno and his coworkers examined the effect of ischaemia/reperfusion in both cyanotic and normoxic models in Langendorff-perfused male Sprague-Dawley rats and reported a worse outcome in the cyanotic group [58] which was ascribed to the sudden increase in oxygen tension in the coronary perfusate. Further, in order to examine the effect during CPB of hyperoxia on distant organs, Fujii et al. [59] examined inflammatory responses at high (400 mmHg) and normal P_a_O_2_ (100–150 mmHg) levels in a rat model of cardiopulmonary bypass. They found an increase in proinflammatory cytokines and total lung water in the hyperoxic group; responses that were suppressed in the normoxic arm.

However, another recent study [60] compared cardiac function in Sprague-Dawley rats following a 25-minute KCl induced cardiac arrest. The rats were resuscitated with 100% oxygen on CPB until 3 minutes after return of spontaneous circulation, at which point they received either 40–50% O_2_ via CPB or 100% O_2_. Postresuscitation haemodynamics, cardiac function, mitochondrial function, and immunostaining of 3-nitrotyrosine were then compared between the two different groups. They found that the hyperoxic group had significantly improved myocardial and mitochondrial function compared to the normoxic group. However, these rats were studied only at 1 hour following ischaemia, in an isolated heart model. Therefore, longer term deleterious effects, damage to end-organs as well as systemic stress, and the interaction of these effects with the myocardium cannot be commented upon.

Whilst suggestive, these studies were however only an ex vivo, small animal studies, so much of the recent work is performed in larger animals, especially swine. Work on large animals included experiments on piglet hearts of less than 4 days of age, perfusing them on cardiopulmonary bypass for 90 minutes with 40% oxygen saturated blood and then increased the saturation to 100% [61]. The workers compared these hearts to a control group, which were treated in an identical fashion, but oxygen saturation was maintained at 100%. They reported a decrease in myocardial function in hearts exposed to hypoxia and reoxygenation with no significant decrease in myocardial function in the control group. In doing so, these findings recapitulated the rodent work, again confirming that transient hypoxia prior to CPB is related to a poorer postoperative outcome. However, these investigations only examined preoperative transient hypoxia models then treated with hyperoxic cardiopulmonary bypass.

In order to address the questions around the most appropriate P_a_O_2_ to maintain on CPB, Ihnken et al. [22] subjected Duroc Yorkshire piglets to cardiopulmonary bypass and blood cardioplegia (BCP). They divided the piglets into control, treatment, and nontreatment groups. Piglets in the control group had 1 hour of CPB without hypoxia with 30 min of BCP arrest. Other piglets were subjected to 120 min of hypoxia (P_a_O_2_ = 20–30 mmHg) before the procedure. Piglets in the nontreatment group were reoxygenated on CPB for 5 min with high P_a_O_2_ (350–450 mmHg) followed by 30 min of BCP arrest and 25 min of reoxygenation/reperfusion on CPB with high P_a_O_2_ levels. Piglets in the treatment group had normoxic CPB (P_a_O_2_ 100 mmHg) and BCP. They reported that hypoxic piglets with hyperoxic CPB developed worse LV function and reoxygenation injury compared to the group treated with normoxic CPB. In addition to this observation, they found that hyperoxic CPB led to a several-fold increase in lipid peroxidation and oxygen consumption as well as a decrease in antioxidant capacity compared to a normoxic group. Thus on both functional grounds and biochemical, they had demonstrated benefit to the myocardium of a normoxic approach to CPB.

Further pursuing the hypothesis that ROS were at least in part responsible for reoxygenation injury, Morita et al. [62] studied 40 immature Yorkshire Duroc piglets of 2 weeks of age. They analysed reduction of nitric oxide (an intermediate here of cytotoxic oxygen species) production with controlled cardiac reoxygenation in acutely hypoxic infantile hearts. They reported that CPB and blood cardioplegic arrest caused no functional or biochemical change in hearts maintained at normoxia. In piglets in which hypoxia was induced on a ventilator prior to surgery, a brief period of abrupt hyperoxic reoxygenation as short as 5 minutes after initiation of CPB, is enough to cause injury and that there was no myocardial protective effect even by controlling the P_a_O_2_ during cardioplegia delivery after this period. However, in a group in which the oxygen tension was slowly increased at the initiation of CPB, this large rise in NO production was not seen nor was there the previously seen elevation of dienes, and functionally the hearts were much closer to the normoxic group. This finding suggests that it is a sudden and abrupt change in the oxygen tension to which the myocardium is exposed, rather than a high P_a_O_2_ *per se*, which may be responsible for at least some of the reoxygenation injury.

Of course, the injurious effect of open heart surgery is not limited to the myocardium. To examine whether normoxic bypass had any effect upon the systemic stress endured perioperatively, Bandali et al. [63], using neonatal Yorkshire pigs, demonstrated that initiation of hyperoxia on CPB for period of 2 hours showed an increase in blood glucose levels by 40% during the first hour and a further increase by 12% in the second hour. Additionally they reported that following 2 hours of normoxic CPB, blood glucose levels remained unchanged. However with the induction of hyperoxic CPB, blood glucose levels raised by 46% and returned to normal following reestablishment of normoxia. So, broadly speaking, normoxic CPB is associated with significantly reduced elevation of blood glucose; this is indicative of reduced flux through the HPA endocrine axis and a decreased level of systemic stress.

Although these studies shed some light on the concept of hypoxia and hyperoxic reoxygenation injury, they had their limitations as the hypoxia was acute and induced by reducing F_i_O_2_, not by right to left sided shunts as would be seen in a cyanotic neonate. These patients will typically be operated upon some months after birth, so the ex utero hypoxic state is more chronic than the acute hypoxia in these models.

In order to address these criticisms and replicate an anatomical shunt causing cyanosis, Silverman and coworkers [64] used a canine model to anastomose left atrium proximal to a banded pulmonary artery to create cyanosis. They then compared this group to dogs with either pulmonary banding alone or no surgical intervention at all. They showed that after 3 months, there was a significant reduction in global biventricular function in the cyanotic group when compared with the other two groups. They later subjected these animals to cardiopulmonary bypass and cardioplegic arrest. They reported that there was a significant reduction in ATP levels in cyanotic dogs, which was preserved in the other groups. They concluded that chronic hypoxia impairs global ventricular function and predisposes to the accelerated depletion of high-energy phosphates during cardioplegic arrest. These findings are similar to those performed earlier using animals exposed to hypoxic environments and confirm the validity of those conclusions.

However, a potentially valid criticism is that cyanosis in these models was interrupted with a period of normoxia after birth. This is clearly a large deviation from the pathophysiological state these models hope to duplicate and limits the applicability of the conclusions.

Addressing these complaints by attempting to produce a model of hypoxia from birth, Baker et al. [65] kept pregnant New Zealand white rabbits in a normoxic environment throughout their study (F_i_O_2_ of 0.21). Once the kits were born, following their first feed, they were immediately transferred to a hypoxic environment (F_i_O_2_ of 0.09). They maintained the oxygen level in the chamber at this level throughout the study. However they transferred the kits back to their mother twice a day for 20 min each to allow feeding. They have indicated that in a preliminary study they observed that mothers that were maintained at (F_i_O_2_ of 0.09) were unable to nurse their offspring. They reported that hearts of hypoxic from birth compared with normoxic controls had better protection against ischaemia in hypothermic conditions, undergoing hypothermia plus cardioplegia. This is surprising, and in conflict with what would be expected based upon both theoretical considerations and the available other clinical and experimental evidence. This model is not a complete replication of cyanotic congenital heart disease, however, as the kits were returned to 21% oxygen regularly for feeding, which is clearly not something which would happen in the human disease state.

In order to address the aberrant finding, Milano et al. [66] examined the effect of daily reoxygenation further to delineate where the apparent benefit was originating. They dubbed this model “chronic hypoxia with daily reoxygenation” (CHReox). In their animal model, they reported that daily reoxygenation during chronic hypoxia reduced ischaemia/reperfusion injury as well as improved recovery of ventricular performance. They concluded that it is not hypoxia in itself that results in protection in these models, but rather a daily reoxygenation associated with chronic hypoxia. This could be due to an increase of NO production in CHReox, which is involved in the ischaemic preconditioning, where short antecedent ischaemic periods render tissue more resistant to subsequent prolonged ischaemic insults. These findings then bring the work of Baker et al. [65] into consistency with other studies.

However, this study is limited by the fact that their animal models were made hypoxic at 5 weeks of age, whereas children with cyanotic congenital heart defects are cyanotic from birth and usually present with ventricular hypertrophy, pressure, and/or volume overload, heart failure, or a combination. Only the cyanotic element is modelled in the aforementioned investigations and none have avoided any normoxic period entirely.

To avoid episodes of normoxic exposure, Corno and coworkers [67] came up with a novel idea of storing animals in specially designed cages that remained hypoxic throughout even during and cleaning and feeding. They randomly allocated eighteen 5-week-old male Sprague-Dawley rats to be exposed to room air (F_i_O_2_ = 0.21) or chronic hypoxia (F_i_O_2_ = 0.10) for 2 weeks. They reported that uncontrolled reoxygenation of hypoxic hearts had negative systemic and cardiac effects. Although this was certainly a novel approach, an important drawback to this study, however, is that this design does not closely model the pathophysiology of clinical syndromes of cyanotic heart disease as the animals were already exposed to 5 weeks of normoxia before being subjected to hypoxia.

So although there are some well-performed studies in animal models, due to either flaws in design or inherent difficulties in reproducing the pathological state, the evidence they produce is only suggestive rather than a convincing demonstration for a deleterious effect from cyanosis and hyperoxic reoxygenation. However, evidence from clinical sources, taken together with the experimental data, strengthens the case.

## 5. Clinical Evidence for Hyperoxic Injury

Some of the earliest data to emerge on questions of comparisons of normoxic to hyperoxic cardiopulmonary bypass in the clinical setting was from Ihnken et al. [68]. They randomised 40 consecutive (adult, acyanotic) patients who were listed for coronary artery bypass graft surgery to either hyperoxic or normoxic CPB. Moderate hypothermia (28° to 32°C) was achieved during CPB in these patients. They reported higher release of creatine kinase, LDH, antioxidant levels, malondialdehyde, and polymorphonuclear elastase in the hyperoxic group. There were also extracardiac improvements in the normoxic group; the hyperoxic patients underwent a 57% longer duration of mechanical ventilation postoperatively, although this did not reach significance. While they reported lower vital capacity and forced vital capacity after CPB in both groups, normoxic patients had better vital capacity and forced vital capacity after CPB than the hyperoxic group. Maximal expiratory flow was also was reduced in hyperoxic patients with no changes in normoxic ones. They concluded that hyperoxia can contribute to multiorgan injury and failure and is unnecessary during established extracorporeal circulation. However, the applicability of this data to the paediatric and in particular the paediatric cyanotic population is clearly limited, although it does suggest that the basic and experimental data may be borne out in the clinical setting.

In the paediatric environ, Modi et al. [4] observed twenty-nine patients with congenital heart disease undergoing cardiac surgery. Of these, twenty were cyanotic and nine were acyanotic. All of these patients underwent at least 30 minutes of hyperoxic CPB before ischaemic cardioplegic arrest. They assessed plasma levels of troponin I (TnI, a marker of myocardial damage) at 1, 10, and 30 minutes of CPB. They reported an increase in troponin I levels with time in both groups; however the rate of increase was greater in the cyanotic population by approximately threefold. Thus this effectively provides clinical evidence of myocardial reoxygenation injury as a direct result of hyperoxic CPB distinct from cardioplegic ischaemia/reperfusion insult; the rises were seen prior to the use of any cardioplegia, as samples were taken before aortic cross clamping. However, this study was purely observational, and so did not provide any further insights into mechanisms or changes in the underlying pathophysiology.

Attempting to take a closer look at the redox state and inflammatory mechanisms that take place in these scenarios, Bulutcu et al. [69] performed a prospective randomised trial where they allocated cyanotic patients to either an F_i_O_2_ on CPB of 1.0 (producing a measured P_a_O_2_ of 300–350 mmHg in their patients) or an F_i_O_2_ of 0.21 (P_a_O_2_: 90–110 mmHg). Furthermore, they also studied an acyanotic group that were at F_i_O_2_ of 1.0. They measured the antioxidant reserve capacity, as well as the levels of TNF-*α* and IL-6. As expected from the earlier discussion of ROS production in cyanosis, they found a lower antioxidant reserve capacity in cyanotic groups when compared to the acyanotic patients at baseline. Following initiation of CPB, TNF-*α* and IL-6 levels in the cyanotic groups were higher than for the acyanotic group. Cyanotic groups with hyperoxic CPB also had further reduced antioxidant reserve capacity compared to cyanotic group with normoxic CPB as well as higher TNF and IL-6 levels. Unfortunately it is not clear from their report what the P_a_O_2_ was during anaesthetic induction as a consequence of the supplemental oxygen administration which occurred, which could certainly have a confounding impact upon the normoxic group.

To further ascertain the underlying mechanisms, we [70] examined transcriptomic changes in a trial of paediatric patients with the tetralogy of Fallot causing cyanosis undergoing corrective cardiac surgery. Patients received either controlled reoxygenation (50–80 mmHg) or hyperoxic/standard CPB (150–180 mmHg). We found significant whole genome expression changes in the hyperoxic group that were not seen with controlled reoxygenation. Our findings suggested a decrease in the transcripts responsible for the adaptive and remodelling capacity of cyanotic hearts subjected to hyperoxia compared with controlled reoxygenation CPB. Our data also showed a reduction in global mRNA levels with hyperoxic CPB, suggesting a detrimental effect of hyperoxic CPB to the myocardium as a result of possible reduction of taurine, which can lead to cardiomyocyte atrophy, mitochondrial and myofibre damage, and cardiac dysfunction.

These studies, however, do not address the questions as to the wider systemic stress and end-organ injuries as outcome measures. Our group [5] performed a randomised trial which compared the effect of normoxic (50–80 mmHg) versus hyperoxic (150–180 mmHg) CPB on cyanotic children undergoing corrective cardiac surgery. We achieved a normoxic CPB by delivering medical nitrogen to the oxygenator and using an in-line PO_2_ monitor to measure the PO_2_ of the prime and matching it to the patient's own (cyanotic) P_a_O_2_ levels. Anaesthetic induction in the normoxic group was carried out with 21% oxygen, whilst in the hyperoxic group the F_i_O_2_ was 50%. We demonstrated that compared to hyperoxic CPB, normoxic CPB was associated with lower end-organ damage; cardiac, liver, and brain injuries were assessed with Tn-I, Alpha GST, and protein S100 and in all three cases were significantly reduced. In addition we reported lower levels of 8-isoprostane, indicative of a lesser degree of reoxygenation injury in the normoxic group. So not only does it seem that normoxic cardiopulmonary bypass is associated with an improved myocardial outcome, it is also correlated with a lesser degree of end-organ injury.

Some have wondered whether there is a third approach between the extremes of using either 100% or 21% oxygen for reoxygenation. Babu et al. [71] compared hyperoxic versus controlled oxygenation in a graded manner during initiation of cardiopulmonary bypass in cyanotic children undergoing cardiac surgery. They achieved controlled reoxygenation by initiating the CPB with an effective F_i_O_2_ of 0.21 and increased it by 0.1 per minute to ultimately achieve an equivalent of 0.6 (PO_2_: 200–300 mmHg) at five minutes. The CPB in the hyperoxic group was started with F_i_O_2_ of 0.6 from the beginning. They reported lower CPK-MB levels and shorter ventilation time in the controlled reoxygenation group compared to hyperoxic CPB. It is noteworthy however that all patients during the anaesthetic induction were ventilated using F_i_O_2_ of 0.6, which may be confounding. In addition, the P_a_O_2_ was unclear from their report when they initiated the CPB with an F_i_O_2_ of 0.21. They indicated that to minimize costs, they opted to not use in-line PO_2_ monitors for instantaneous PO_2_ measurement and instead they increased oxygen levels over a 5-minute period [64, 71]. So there are significant limitations to this study; however, this novel approach may go some way to ameliorate concerns about the safety of purely normoxic CPB. It is also strongly reminiscent of the basic work of Morita et al. [62] who also took a graded approach to reoxygenation in animal models and found results similar to a normoxic group.

In support of these findings Joachimsson et al. [72] argue that in clinical practice, hyperoxic CPB is likely never needed, since a P_a_O_2_ of 400 to 500 mmHg produces only a trivial increase in oxygen content compared with a P_a_O_2_ of 100 to 150 mmHg, which is easily achieved using a modern oxygenator [73] and that in both instances the oxygen saturation is essentially 100%. Moreover, a CPB P_a_O_2_ of more than 180 mmHg has been associated with impairment of peripheral perfusion. Furthermore, an increased incidence of ventricular fibrillation has been reported [74] as well as some findings that hyperoxia may cause cerebral vasoconstriction that leads to nonhomogeneous oxygen distribution [75] and regional hypoperfusion.

Most of these studies from clinical practice have regarded cyanotic heart disease as one distinct entity. In reality, it consists of a large number of syndromes, many with cardiac and extracardiac manifestations. We have attempted to break down the benefits of normoxic cardiopulmonary bypass further by examining the effects differentially on patients with univentricular or biventricular circulations [76]. We examined markers of cardiac, hepatic, and cerebral injury, as well as long term functional outcome. We demonstrated a significant improvement in both in markers of organ damage and in oxidative stress, a lower degree of systemic injury, as well as a significantly improved outcome at 7 years postoperatively in univentricular patients undergoing open heart surgery for congenital heart disease. There is however a paucity of evidence considering the univentricular circulation so it remains difficult to place this interesting and novel data into context. Additionally, we also showed a significant reduction in the level of postoperative cortisol and proinflammatory cytokines in both biventricular and univentricular circulations; the proinflammatory cascade consequent to CPB as well as the surgical systemic inflammatory response is accountable for end-organ dysfunction postoperatively. Therefore, any reduction in inflammatory mediators as a consequence of this intervention may lead to an improvement in postoperative systemic dysfunction and possibly overall outcome measures.

Considering contraindications to normoxic CPB, the only significant cautions have come from Nollert et al. [77]. They have provided data that show that normoxic CPB should not be considered during deep hypothermic circulatory arrest (DHCA) which can lead to cerebrals insult (assessed by protein S100 and histological evaluation), which showed cerebral insults in particular to neocortex and hippocampal region. In the converse situation, Fontes et al. [78] examined whether hyperoxia might contribute to cognitive decline following cardiopulmonary bypass; no association was found. These findings were confirmed experimentally by Wang and colleagues in 2012 [79], who found that hyperoxic management during DHCA demonstrated better cerebral protection than normoxic.

So DHCA remains the strongest indication for conventional oxygenation on CPB; for the remainder, there is strong experimental and clinical evidence of benefit to a controlled reoxygenation strategy.

## 6. Conclusion

It has been demonstrated that hyperoxic cardiopulmonary bypass predisposes towards direct myocardial injury and systemic stress, with no counterbalancing benefit. In fact not only is this unfavourable effect apparent in the cyanotic population, but there are also reports that even in acyanotic conditions; controlled reoxygenation can be advantageous. Normoxic cardiopulmonary bypass has been reported to be safe and easy to achieve, as long as the appropriate equipment is available, and the perfusionist and anaesthetist are both familiar with the technique. Despite many pieces of both clinical and experimental evidence that hyperoxic CPB can have a detrimental effect on the cyanotic population it seems that the use of controlled reoxygenation is not widely accepted. It is our hope that this presentation of the available evidence might prompt a reevaluation of the wisdom of this approach. Given the limited numbers of patients presenting to each centre, and the variation in current practice, it seems as though a large prospective randomised control trial would be the best way to obtain a definitive answer to the questions raised herein. Certainly, the potential benefits are likely to be significant.
